# 3D Printed Biomimetic PCL Scaffold as Framework Interspersed With Collagen for Long Segment Tracheal Replacement

**DOI:** 10.3389/fcell.2021.629796

**Published:** 2021-01-21

**Authors:** Yunlang She, Ziwen Fan, Long Wang, Yinze Li, Weiyan Sun, Hai Tang, Lei Zhang, Liang Wu, Hui Zheng, Chang Chen

**Affiliations:** Department of Thoracic Surgery, Shanghai Pulmonary Hospital, Tongji University School of Medicine, Shanghai, China

**Keywords:** biomimetic trachea development, ring structure, 3D-printing, tracheal replacement, mechanical property

## Abstract

The rapid development of tissue engineering technology has provided new methods for tracheal replacement. However, none of the previously developed biomimetic tracheas exhibit both the anatomy (separated-ring structure) and mechanical behavior (radial rigidity and longitudinal flexibility) mimicking those of native trachea, which greatly restricts their clinical application. Herein, we proposed a biomimetic scaffold with a separated-ring structure: a polycaprolactone (PCL) scaffold with a ring-hollow alternating structure was three-dimensionally printed as a framework, and collagen sponge was embedded in the hollows amid the PCL rings by pouring followed by lyophilization. The biomimetic scaffold exhibited bionic radial rigidity based on compressive tests and longitudinal flexibility based on three-point bending tests. Furthermore, the biomimetic scaffold was recolonized by chondrocytes and developed tracheal cartilage *in vitro*. *In vivo* experiments showed substantial deposition of tracheal cartilage and formation of a biomimetic trachea mimicking the native trachea both structurally and mechanically. Finally, a long-segment tracheal replacement experiment in a rabbit model showed that the engineered biomimetic trachea elicited a satisfactory repair outcome. These results highlight the advantage of a biomimetic trachea with a separated-ring structure that mimics the native trachea both structurally and mechanically and demonstrates its promise in repairing long-segment tracheal defects.

## Introduction

The trachea is a hollow organ that plays a vital role in respiration and indirect roles in swallowing and speech (Udelsman et al., 2018). Tracheal defects are usually caused by congenital anomalies, tumor ablation, and traumatic injuries and can significantly affect a patient’s quality of life. However, in the case of long-segment tracheal defects (defects spanning 50% of the total tracheal length in adults or about 30% in children), it is almost impossible to reconstruct the defect using the gold-standard treatment of end-to-end anastomosis (Li et al., 2019). Consequently, investigating tracheal substitutes that can be implanted to reconstruct a continuous trachea are being developed (Best et al., 2018), such as cell-free prostheses, allografts, and autografts. Despite the broad range of tracheal substitutes available, each has its own inherent drawbacks (Dikina et al., 2018). Cell-free prostheses have met with limited success as they induce hemorrhaging and luminal stenosis and are prone to dislocation. Allografts are associated with the insurmountable shortcomings of immune rejection and a shortage of donors. While autografts are not susceptible to rejection, their shape and size are not ideal for tracheal replacement (Nomoto et al., 2013).

Recently, tissue engineering technologies based on combinations of autologous cells and biomaterials have been pursued to develop an ideal tracheal substitute (Park et al., 2019; Dharmadhikari et al., 2020). However, the clinical performance of tissue-engineered tracheal substitutes has yet to meet its theoretical potential, which is mainly ascribed to their inadequate anatomy and function (Mansfield et al., 2016). The native trachea comprises multiple cartilage rings alternating with connective tissue rings. The cartilage rings provide radial rigidity and prevent the collapse of the tracheal lumen during respiratory movements, while the connective tissue rings permit longitudinal flexibility and mobility to ensure stability even during rotation, flexion, and extension of the neck (Park et al., 2015). However, most of the proposed designs for tracheal substitutes constitute a simple tube comprising a single continuous cartilage tissue section, which fails to mimic the natural ring structure (Luo et al., 2013; Xu et al., 2017, 2020a). Previous approaches have placed too much emphasis on the radial rigidity and overlooked the longitudinal flexibility. Therefore, a biomimetic trachea with biomimetic structural and mechanical properties is urgently needed to repair tracheal defects.

Three-dimensional (3D) printing of structures derived by computed tomography image or computer-aided design is a promising technique for the precise fabrication of tracheal substitutes that match the individual composition, mechanical properties, and 3D architecture of a patient’s trachea (Xia et al., 2019). Polycaprolactone (PCL), which is approved by the United States Food and Drug Administration (FDA) for internal use in the human body, is the most widely used 3D-printable biomaterial (Li et al., 2020). PCL has excellent biocompatibility and suitable mechanical properties for use in 3D-printing of a tracheal scaffold (Gao et al., 2017; Townsend et al., 2018). Recently, Ke et al. (2019) 3D-printed a patient-matched tracheal ring using PCL and human mesenchymal stem cell-laden hydrogels, however, a troublesome procedure is still needed to incorporate those single rings into an integrated tracheal tube. Theoretically, it is feasible to 3D-print a biomimetic PCL scaffold with a separated-ring structure to closely mimic the native tracheal anatomy. However, PCL is hydrophobic, which is unfavorable for cell affinity and tracheal cartilage formation. Collagen, as the main component of cartilage tissue, is highly hydrophilic and, thus, is promising to counterbalance the drawbacks of PCL in composite materials (Xu et al., 2019). Based on the properties of these materials, a PCL-collagen composite scaffold is expected to provide both cell affinity and mechanical stability.

**SCHEME 1:**
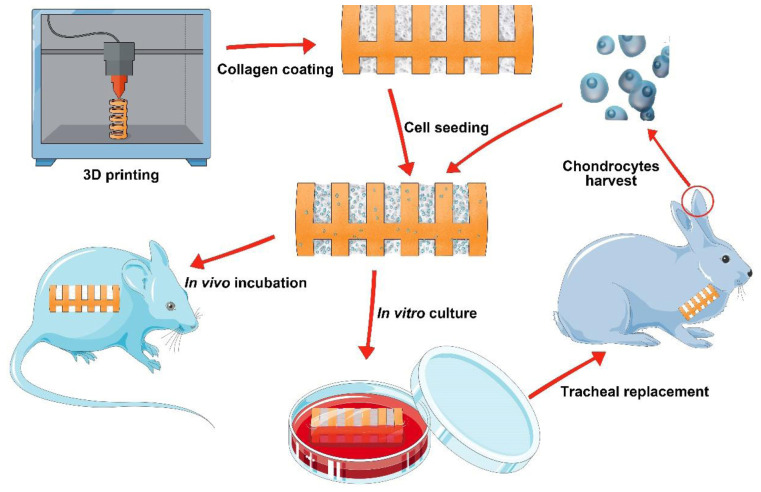
The schematic of the overall experiment.

Herein, we conceived a biomimetic scaffold with a separated-ring structure. A PCL scaffold with a ring-hollow alternating structure was 3D-printed as a framework, and collagen sponge was embedded onto the hollows amid the PCL rings by pouring-lyophilization method. The biomimetic scaffold was then seeded with chondrocytes and incubated either *in vitro* or *in vivo* to generate a biomimetic trachea that mimics both the structure and mechanical properties of the native trachea. Finally, the feasibility of using this biomimetic trachea to repair long-segment tracheal defects was investigated in a rabbit model. The overall experiment was illustrated as Scheme 1.

## Materials and Methods

### 3D-Printed Biomimetic PCL Scaffold

The 3D-printed biomimetic PCL scaffold with a separated-ring structure was designed to mimic the anatomy of the native trachea in a 4-month-old New Zealand white rabbit. PCL particles (Mn = ∼60,000, Purac Biochem, the Netherlands) were fabricated by a 3D-printer (FoChif Tech, Shanghai, China) based on the principle of fused deposition molding (Xia et al., 2019). Briefly, the PCL particles were melt-extruded at 110°C onto a substrate along a predesigned printing path to generate a biomimetic PCL framework with ring-hollow alternating structure, during which a C-shaped photosensitive resin was prefabricated by 3D printing technology to occupy the hollows amid the PCL rings. The hollows were formed after the remove of C-shaped photosensitive resin following the 3D-printing procedure. Besides, a continuous PCL bar (1.5 mm in width) was designed as a backbone to integrate the separated rings. The printed biomimetic PCL scaffold comprised rings 1.0 mm wide with a 5.0-mm inner luminal diameter and a 7.0-mm outer luminal diameter. In addition, a cylindrical PCL tube with the same luminal diameter was printed using the same methods.

The longitudinal flexibility of the biomimetic PCL scaffold and its cylindrical PCL tube counterpart were initial roughly estimated by manipulating it with tweezers and hands. The longitudinal flexibility was further evaluated through a three-point bending test, and the lateral rigidity was evaluated through a radial compression test (Instron, Norwood, MA, United States) with a 2-kN load cell. The force and displacement were monitored during deformation at a constant crosshead velocity of 0.5 mm/min. In three-point bending test, the distance between two lower contact points was adjusted so that the load was applied to the PCL ring at both ends of the scaffold, and a maximum three-point bending displacement of 3.5 mm was determined to achieve a bending angle of 45°. In the compression test, the scaffold kept horizontal orientation, and maximum radial compression displacement of 5.0 mm was adopted to achieve full deformation of the scaffold.

### Collagen Embedding

The biomimetic PCL scaffold and cylindrical PCL tube were inserted between two silicone tubes: the outer diameter of the inner silicone tube was 4.5 mm, and the inner diameter of the outer silicon tube was 7.5 mm. Then, a solution of 10 mg/mL type II collagen (Sigma) in 0.1 M acetic acid was injected into the gap between the two silicone tubes. The whole scaffold was frozen at −20°C for 24 h and was lyophilized until it was dry. The lyophilized scaffold was crosslinked with an EDC/NHS solution at −4°C for 24 h, as described previously (Xu et al., 2019). The scaffold was rinsed with deionized water to remove the unreacted EDC/NHS and lyophilized again to produce the biomimetic scaffold and cylindrical tube.

### Scanning Electron Microscopy

The biomimetic scaffold and cylindrical tube were sputter-coated with gold for 50 s to increase their conductivity. The morphology of each structure was analyzed by Scanning electron microscopy (SEM) (Hitachi TM-1000, Japan) at an accelerating voltage of 15 kV.

### Isolation and Culture of Chondrocytes

All animal experiments were approved by the Shanghai Pulmonary Hospital Ethics Committee. Six 2-month-old New Zealand white rabbits with an average weight of 3.0 kg were purchased from the Shanghai Jiagan Breeding Factory (Shanghai, China). Auricular cartilage samples were harvested from the New Zealand white rabbits, minced into approximately 1.0-mm^3^ pieces, and digested for 8 h in 0.15% type II collagenase (Gibco) in Dulbecco’s Modified Eagle Medium (DMEM, Gibco) at 37°C. Isolated chondrocytes were cultured in DMEM supplemented with 10% fetal bovine serum (FBS, Gibco) and 1% penicillin-streptomycin at 37°C in 5% CO_2_ (Xu et al., 2019). The cells were used at passage two (P2) for *in vitro* and *in vivo* tracheal cartilage formation.

### *In vitro* Tracheal Cartilage Formation

Chondrocytes were harvested at P2, resuspended at a density of 5.0 × 10^7^ cells/mL, and a total of 1 mL chondrocytes suspension was loaded evenly onto the biomimetic scaffolds. The cell-scaffold constructs were incubated at 37°C for 4 h and cultured in DMEM containing 10% FBS at 37°C under 5% CO_2_. The medium was changed every 2 days. The constructs were submerged in the medium during the whole culture course. We changed the culture media every 2 days with a roll-over to ensure homogeneous nutrient penetration and continuously cultured *in vitro* for 4 weeks then harvested for gross and histological examinations as well as biochemical quantitative analyses.

### *In vivo* Incubation

Six 1-month-old Balb/c nude mice were obtained from Shanghai Slaccas Experimental Animal Ltd. Each animal was anesthetized with 0.3 mL of 1% pentobarbital sodium; following aseptic preparation on their back, the skin was incised, and the subcutaneous tissue was separated to form a pocket. The cell-scaffold constructs prepared as described above was placed directly into the pocket without pre-culture *in vitro*. The incision was then closed, and the animal was allowed to recover from anesthesia.

After the constructs were incubated in nude mice for 6 weeks, the biomimetic trachea samples were retrieved from these mice, and three of which were used for gross and histological evaluation, the other three samples were used for biochemical and biomechanical (including three-point bending and radial compression) examinations.

### Tracheal Replacement

Six rabbits were anesthetized by intravenous injection of pentobarbital sodium (30 mg/kg). Under sterile conditions, a midline incision was made in the anterior neck of each animal. The surrounding tissue was separated carefully to expose the trachea. An approximately 1.0-cm segment of the trachea was excised, starting 1.5 cm below the cricoid cartilage. The chondrocytes-scaffold construct prepared as described above was pre-cultured *in vitro* for 5 days and then placed into the defect, and end-to-end continuous anastomosis was performed with 6–0 absorbable sutures. The wound was closed in layers with 4–0 sutures. The rabbits recovered while being treated with antibiotics for 7 days to prevent infection.

To evaluation the luminal conditions, bronchoscopy examinations were performed under anesthesia 4 and 8 weeks after surgery using a distal chip flexible endoscope system (Olympus, Tokyo, Japan).

### Histological and Immunohistochemical Staining

Samples were fixed in 4% paraformaldehyde, embedded in paraffin, and sectioned for histological and immunohistochemical analysis. Hematoxylin & eosin (HE), safranin-O, toluidine blue staining were performed to evaluate the engineered samples. Staining of type II collagen was also conducted to further confirm a cartilage-specific phenotype according to a previously established method (Xu et al., 2019, 2020c).

### Biochemical Analyses

The constructs that were subcutaneously incubated in nude mice were collected and minced to conduct cartilage-related biochemical evaluations for DNA content, glycosaminoglycan (GAG) content, and total collagen content using a PicoGreen dsDNA assay (Invitrogen), dimethylmethylene blue assay (Sigma-Aldrich), and hydroxyproline assay kit (Sigma-Aldrich), respectively. The type II collagen content in each sample was quantified by an enzyme-linked immunosorbent assay, as described previously (Zhou et al., 2018).

### Statistical Analyses

All experimental data were expressed as the mean ± standard deviation from at least three experiments. Data were analyzed using GraphPad Prism 5.0 by one-way ANOVA, and differences with *P* < 0.05 were considered statistically significant.

## Results

### Biomimetic Scaffold Preparation

**FIGURE 1:**
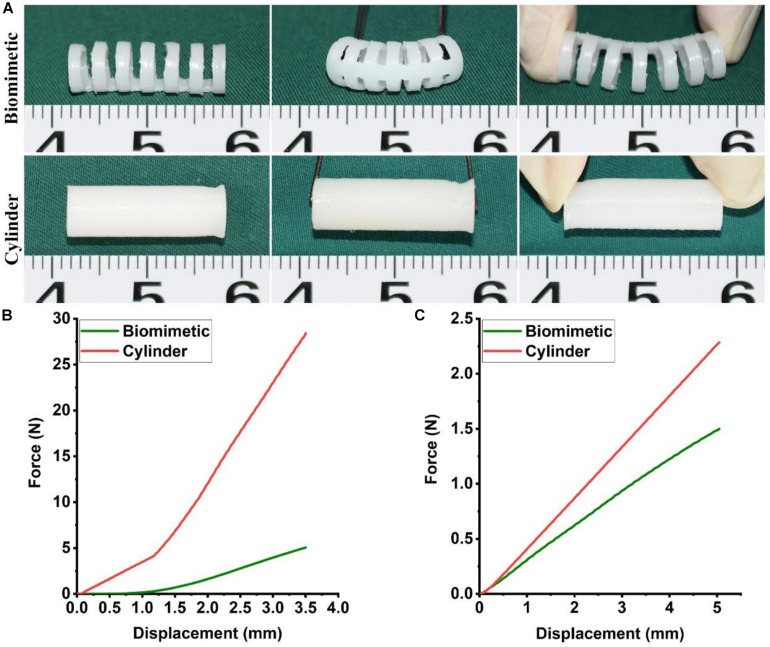
Mechanical properties of the biomimetic PCL scaffold. Bending and extending tests of the biomimetic PCL scaffold and the cylindrical PCL tube **(A)**. Three-point bending **(B)** and compression test **(C)** of the biomimetic PCL scaffold and the cylindrical PCL tube (*N* = 3 per group).

The biomimetic PCL scaffold was designed to closely mimic the anatomy and mechanical behavior of the native trachea in a 4-month-old New Zealand white rabbit. PCL was 3D-printed into a biomimetic framework with a separated-ring structure. The width of each PCL ring was 1.0 mm and the distance between adjacent PCL rings was 1.0 mm. The luminal diameter was 5 mm, which is close to the average diameter of a rabbit’s native trachea (Figure 1A). Mechanical testing results indicated that the biomimetic PCL scaffold exhibited better bending and extension properties in the longitudinal compared with the cylindrical PCL tube (Figure 1A and Supplementary Video 1). Furthermore, the biomimetic PCL scaffold exhibited better longitudinal flexibility in the three-point bending test (Figure 1B), but it maintained similar radial rigidity compared with the cylindrical PCL tube (Figure 1C), and it returned to its original shape when the load was removed (Supplementary Video 2).

**FIGURE 2:**
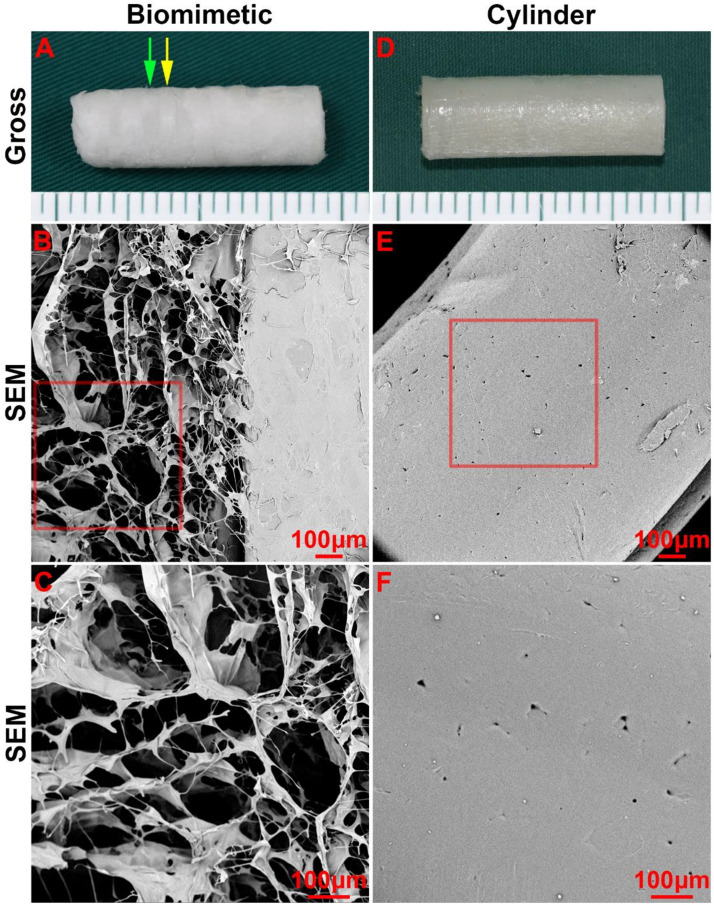
Morphology of the biomimetic scaffold and cylindrical tube. Macro-morphology of the biomimetic scaffold **(A)** and cylindrical tube **(B)**. Micro-morphology of the biomimetic scaffold **(B–C)** and cylindrical tube **(E–F)** as observed by SEM. The green arrow indicates the collagen region, and the yellow arrow indicates the PCL region.

Next, collagen sponge was added into the spaces amid the PCL rings of the scaffold by a pouring-lyophilization method for tracheal cartilage formation. The collagen sponge was successfully loaded between the PCL rings and formed a biomimetic scaffold with a collagen ring-PCL ring alternating structure (Figure 2A). SEM images revealed that the collagen region exhibited a porous structure (Figures 2B,C), which favors chondrocyte attachment, proliferation, and secretion of cartilage-specific extracellular matrix (ECM). Conversely, the PCL regions in both the biomimetic scaffold and cylindrical tube displayed a non-porous structure (Figures 2D–F), which prevented cells from penetrating into the PCL scaffold.

### Tracheal Cartilage Formation *in vitro*

**FIGURE 3:**
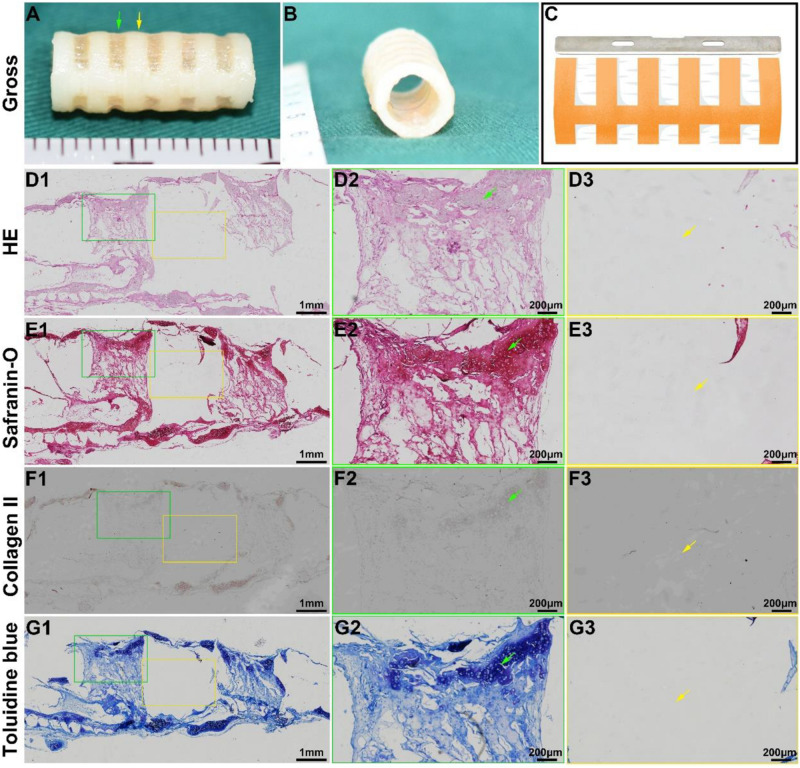
*In vitro* biomimetic trachea regeneration. Gross view of the biomimetic trachea after 4 weeks *in vitro* culture **(A,B)**. **(C)** shows the sectioning schematic. HE, safranin-O, collagen II, and toluidine blue staining of a longitudinal section of the engineered biomimetic trachea **(D1–G1)**. Magnified views of a cartilage ring **(D2–G2)** and PCL ring **(D3–G3)**. The green arrows indicate cartilage regions, and the yellow arrows indicate PCL regions.

Tracheal cartilage formation was evaluated by *in vitro* experiments. After the biomimetic scaffolds were recolonized with chondrocytes and cultured *in vitro* for 4 weeks, the chondrocyte-scaffold constructs formed a tubular structure with a distinguished separated-ring structure (Figures 3A,B). The regions in collagen ring displayed a visible cartilage-like tissue, while the cartilage-like tissue was obscure in the PCL regions. Histological analysis of a longitudinal section further confirmed the marked difference between the collagen region and the PCL region (Figures 3D1–G1): cartilage-like tissue with a primary lacuna structure containing GAG and collagen II was formed in the collagen region (Figures 3D2–G2), while virtually no cartilage tissue was formed in the PCL region (Figures 3D3–G3), and only a thin layer of cartilage-like tissue was observed on the outer surface of the PCL.

### Biomimetic Trachea Formation *in vivo*

**FIGURE 4:**
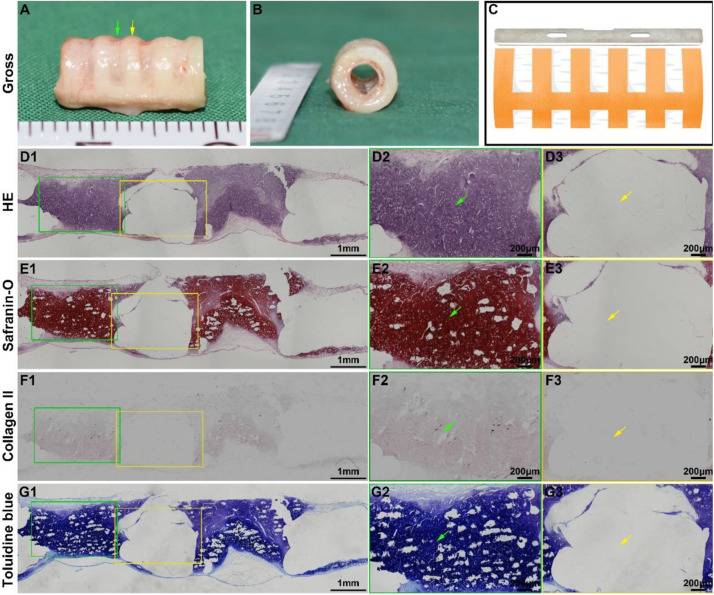
*In vivo* biomimetic trachea formation and histological analysis of a longitudinal section. Gross view of the biomimetic trachea after 6 weeks *in vivo* incubation **(A,B)**. **(C)** shows the sectioning schematic. HE, safranin-O, collagen II, and toluidine blue staining of a longitudinal section of the engineered biomimetic trachea **(D1–G1)**. Magnified views of the cartilage ring **(D2–G2)** and PCL ring **(D3–G3)**. The green arrows indicate cartilage regions, and the yellow arrows indicate PCL regions.

**FIGURE 5:**
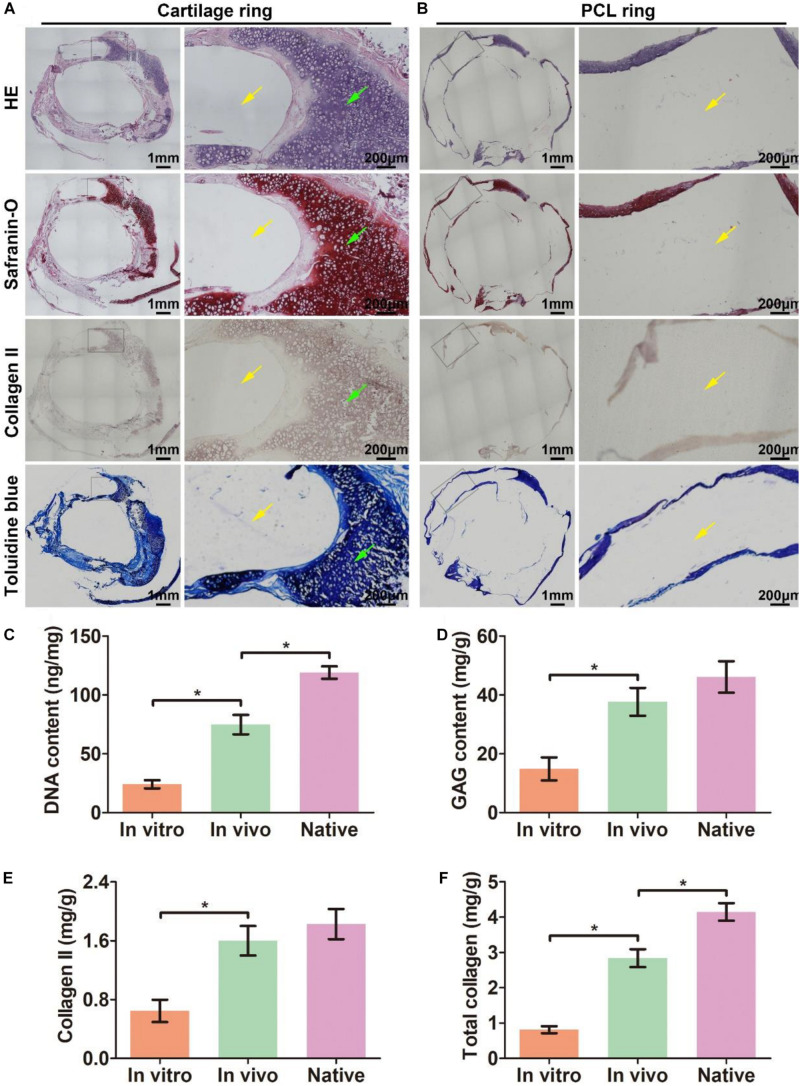
Histological analysis of a transverse section of *in vivo* engineered biomimetic trachea and biochemically quantitative evaluation. HE, safranin-O, collagen II, and toluidine blue staining of a transverse section of the *in vivo* engineered biomimetic trachea and representative magnified images of the cartilage ring **(A)**. HE, safranin-O, collagen II, and toluidine blue staining of a transverse section of the *in vivo* engineered trachea and representative magnified images of the PCL ring **(B)**. The green arrows indicate cartilage regions, and the yellow arrows indicate PCL regions. Quantitative measures of the DNA content **(C)**, GAG content **(D)**, collagen II content **(E)**, and total collagen content **(F)** in samples engineered *in vitro* and *in vivo* as well as its native tracheal counterpart. *N* = 3 per group. *P < 0.05.

The biomimetic scaffold was further evaluated for its ability to facilitate the formation of a biomimetic trachea *in vivo*. After the scaffold was subcutaneously incubated in a nude mouse for 6 weeks, an intact tubular tissue with a visible separated-ring structure was generated (Figures 4A,B). Histological images of a longitudinal section (Figure 4C) showed that cartilage rings and PCL rings were alternately arranged in the structure (Figures 4D1–G1). Specifically, a mature cartilage-specific tissue with a typical lacuna structure and positive staining of safranin-O and collagen II formed in the collagen regions (Figures 4D2–G2), but there were no traces of any cells or tissues in the PCL region (Figures 4D3–G3). These findings were further confirmed by microscopic observation of transverse histological sections (Figures 5A,B). Importantly, quantitative analyses showed that the *in vivo* engineered sample showed apparently higher biochemical contents (DNA, GAG, collagen II, and total collagen) than those of the *in vitro* engineered sample (Figures 5C–F), and had comparable GAG and collagen II contents (Figures 5D,E) and slightly lower DNA and total collagen contents (Figures 5C,F) compared with native tracheal tissue.

**FIGURE 6:**
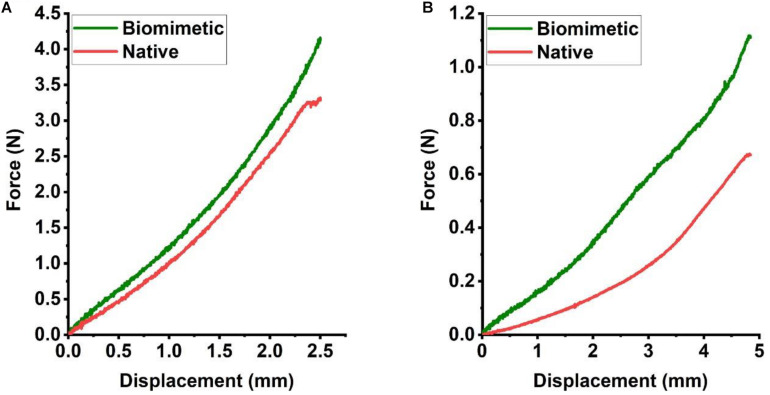
Mechanical characteristics of the engineered biomimetic trachea cultured *in vivo*. Three-point bending **(A)** and compression testing **(B)** of the biomimetic trachea and native trachea (*N* = 3 per group).

The constructs retrieved after 6 weeks of *in vivo* incubation were subjected to mechanical testing, and load-displacement curves were derived to compare the mechanical behavior with that of a native trachea. In a three-point bending test, the load curve of the biomimetic trachea was very similar to that of the native trachea until the displacement reached about 2.5 mm (Figure 6A). This finding indicates that the biomimetic trachea closely mimics the native trachea in terms of longitudinal flexibility. In a radial compression test, the load curve of the biomimetic trachea was discernibly higher than that of the native trachea (Figure 6B), and the biomimetic trachea returned to its original shape when the load was removed (Supplementary Video 3). These findings demonstrated that the biomimetic trachea was superior to the native trachea in terms of radial rigidity.

### Long-Segment Tracheal Replacement Using the Engineered Biomimetic Trachea

**FIGURE 7:**
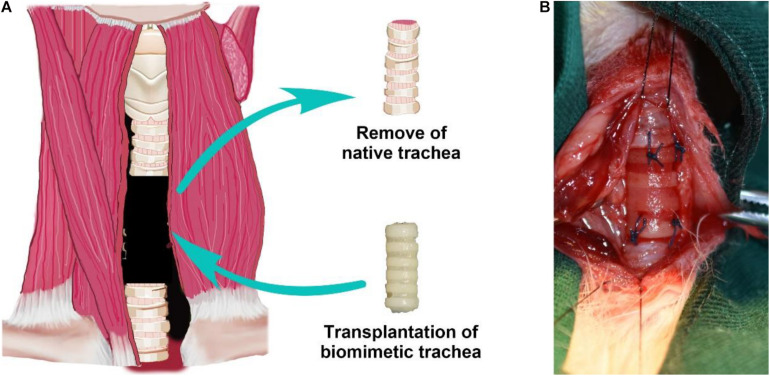
Tracheal replacement using the biomimetic trachea. Schematic illustration of the replacement surgery **(A)**. Biomimetic trachea transplantation by end-to-end anastomosis **(B)**.

To further evaluate the feasibility of the biomimetic trachea in repairing long-segment tracheal defects in a more applied manner, we performed a trachea replacement in a rabbit model (Figure 7). The engineered biomimetic trachea exhibited sufficient strength to be attached to the native trachea using sutures. As listed in the Supplementary Table 1, neither operative death nor anastomotic dehiscence occurred in any rabbit. After the replacement operation, neither air leakage nor collapse around the biomimetic trachea was observed. None of the rabbits showed any sign of stridor or dyspnea during the recovery period. In addition, all rabbits survived without obvious inflammatory exudation, formation of granulation tissues, anastomotic leakage, or stenosis in the operation area over 8 weeks following the surgery.

**FIGURE 8:**
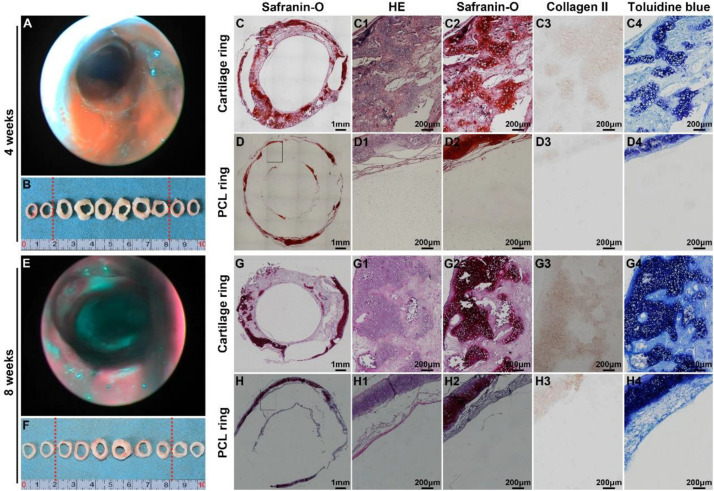
Therapeutic outcome after tracheal replacement. Bronchoscopy and gross examination images of the biomimetic trachea 4 weeks after the replacement surgery **(A,B)**. Histological images of a transverse section of the biomimetic trachea showing a cartilage ring **(C,C1–C4)** and a PCL ring **(D,D1–D4)** 4 weeks after the replacement surgery. Bronchoscopy and gross examination images of the biomimetic trachea 8 weeks after the replacement surgery **(E,F)**. Histological images of a transverse section of the biomimetic trachea showing a cartilage ring **(G,G1–G4)** and a PCL ring **(H,H1–H4)** 8 weeks after the replacement surgery. The biomimetic trachea is delineated by red dotted lines.

**FIGURE 9:**
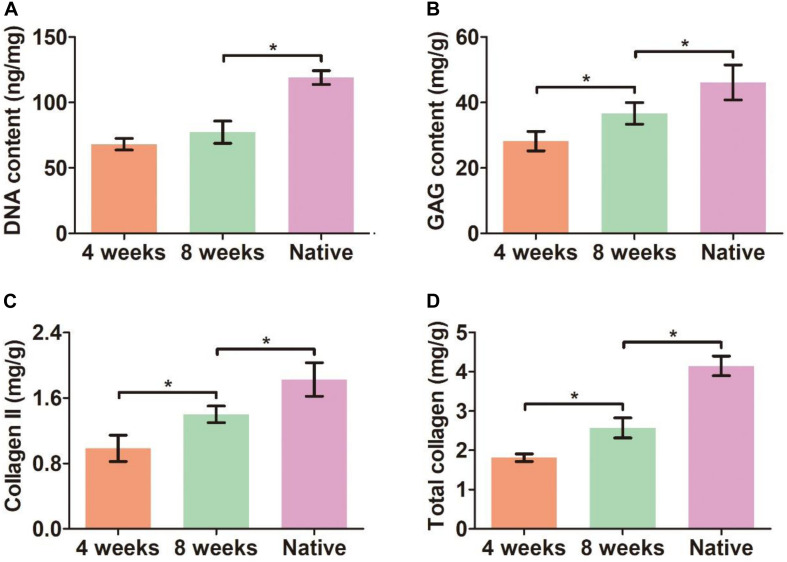
Quantitative analyses of the biomimetic trachea after tracheal replacement. DNA content **(A)**, GAG content **(B)**, collagen II content **(C)**, and total collagen content **(D)** of the biomimetic after tracheal replacement for 4 and 8 weeks. *N* = 3 per group. *P < 0.05.

Bronchoscopy examinations at 4 and 8 weeks following implantation showed that the lumen of the biomimetic trachea remained patent at both time points, and overgrowth of granulation tissue seldom occurred (Figures 8A,E). These findings were further confirmed by gross observation (Figures 8B,F). In addition, histological images showed that the ring shapes in both the cartilage and PCL regions remained intact (Figures 8C,D,G,H). Notably, the biomimetic tracheas developed the structures and features of cartilage-like tissue with lacunar structures (Figures 8C1–D1 and G1–H1) and cartilage ECM, as evidenced by positive safranin-O (Figures 8C2–D2 and G2–H2), immunohistochemical collagen II (Figures 8C3–D3 and G3–H3), and toluidine blue (Figures 8C4–D4 and G4–H4) staining, at both 4 and 8 weeks following implantation. All the quantitative indexes, including contents of DNA, GAG, collagen II, and total collagen, apparently increased with the prolonged tracheal replacement time from 4 to 8 weeks (Figure 9). These results indicate that the biomimetic trachea is suitable for long-segment tracheal replacement.

## Discussion

Developing an ideal tracheal replacement is a great challenge in repairing long-segment tracheal defects (Bogan et al., 2016; Wang et al., 2020). Although tissue engineering technology shows great potential, until now, no substantial breakthrough has been made in creating a biomimetic trachea that mimics the native trachea both structurally and mechanically (Machino et al., 2019). In the current study, we developed a biomimetic scaffold with a separated-ring structure that closely mimics the anatomical structure and mechanical behavior of the native trachea. To achieve this objective, we designed a 3D-printed ring-shaped PCL framework, and ring-shaped porous collagen sponge structures were loaded amid spaced PCL rings. The collagen rings were readily colonized by chondrocytes, and the biomimetic scaffold supported tracheal cartilage formation both *in vitro* and *in vivo*. Furthermore, the engineered biomimetic trachea was successfully used to repair a long-segment tracheal defect in a rabbit model, demonstrating the potential for the clinical translation of this tissue-engineered biomimetic trachea.

As the trachea is a hollow tube, it is often thought that it would be straightforward to recreate its structure *de novo*. However, this apparent simplicity has proven to be deceptive (Den Hondt et al., 2017). It has become increasingly clear from both pre-clinical and clinical studies that further optimization of the construction approach is required (Weiss et al., 2014). Most literature recognizes the importance of tracheal lateral rigidity for normal respiration and ventilation, but they do not fully consider the tracheal longitudinal flexibility, which is needed to ensure the stability of the trachea during rotation, flexion, and extension of the neck (Johnson et al., 2016; Xu et al., 2020a). If a tracheal scaffold lacks the required longitudinal flexibility, the scaffold will become distorted, and the tracheal lumen will narrow during rotation, and the scaffold could tear during flexion and extension (Xu et al., 2020b). Hence, a new design mimicking the native tracheal anatomy could ensure the bionic function of a tracheal substitute (Dikina et al., 2018). The native trachea mainly comprises multiple cartilage rings that are interspersed with connective tissue rings (Bogan et al., 2016). The cartilage rings provide lateral rigidity to maintain airway patency during respiration, while the connective tissue ring provides longitudinal flexibility. Therefore, the ideal tracheal replacement would be characterized by a separated-ring structure to simultaneously mimic the structure and mechanical properties of the native trachea. Recently, a patient-matched tracheal ring was 3D-printed using PCL and human mesenchymal stem cell -laden hydrogels (Ke et al., 2019). Although the 3D-printed tracheal ring showed comparable elastic modulus and yield stress compared to native tracheal ring, it is still faced with the troublesome of integrating those separate rings into long-segment tracheal tissue. In the current study, we designed a biomimetic trachea with such a structure by first 3D-printing a PCL scaffold with a ring-hollow alternating structure as a framework then embedding collagen sponge onto the hollows amid the PCL rings by a pouring-lyophilization method. Mechanical testing demonstrated that the engineered biomimetic trachea exhibited comparable lateral rigidity and longitudinal flexibility to a native rabbit trachea. Thus, the proposed biomimetic scaffold is expected to be a suitable candidate for a biomimetic trachea formation.

Combining scaffold materials could be useful to fabricate a tracheal construct, as one material could provide mechanical integrity to the construct, and the other material could serve as a biocompatible and chondrogenic environment for cells, thus giving rise to a biomimetic design. PCL has been extensively implemented as a 3D-printing material because it is FDA approved, readily printable, and biodegradable (Li et al., 2020). PCL also has sufficient mechanical properties to serve as a tracheal substitute and a slow rate of biodegradation *in vivo*, which can prevent problems associated with rapid degradation, such as postoperative tracheal softening, collapse, and re-stenosis (Ghorbani et al., 2017). Based on these properties, PCL has been widely used in 3D-printing for tracheal tissue engineering applications (Ke et al., 2019). However, the hydrophobicity and lack of porosity of PCL severely hinder its application in tracheal cartilage formation. Collagen, a component of cartilage ECM, is known to induce cell detachment, proliferation, and chondrogenesis (Omori et al., 2008). A previous study showed that a scaffold material coated with collagen sponge provided an improved environment for tracheal tissue regeneration (Xu et al., 2019). Consequently, the PCL scaffold exhibited inferior cell affinity whereas the collagen scaffold displayed superior cell affinity (Lin et al., 2020). Our results demonstrated that the robust cartilage tissue was generated within the collagen ring but only a thin layer of cartilage tissue was observed on the surface of the PCL ring. We combined PCL and collagen and leveraged the difference between each scaffold, to concurrently mimic the mechanical properties of the native trachea and induce biomimetic anatomy formation featured with cartilage rings alternate interspersed with PCL rings structure.

Good biocompatibility and suitable biodegradability are prerequisite for a scaffold to be used in tracheal tissue engineering. Previous extensive studies have demonstrated that both PCL and collagen are biocompatible (Fu et al., 2015; Jia et al., 2020; Nakamura et al., 2020; Semitela et al., 2020), so we did not specifically test the biocompatibility of the composite using a cell proliferation assay (e.g., a CCK-8 kit) or cell viability assay (e.g., live–dead staining). Nevertheless, our results indicated that the biomimetic scaffold comprised of PCL and collagen support tracheal cartilage formation both *in vitro* and *in vivo*, which indirectly indicates that the 3D-printed PCL scaffold with embedded collagen exhibited satisfactory biocompatibility. In addition, we also fully leveraged the contrasting biodegradability between collagen and PCL, in which the collagen displayed relative higher degradable rate (complete degradation *in vivo* for approximate 2–4 months) and the PCL owned extremely low degradable rate (complete degradation *in vivo* requires 2–4 years) (Zhou et al., 2018; Si et al., 2019). The collagen exhibited appropriate degradation rate to match the formation rate of neocartilage tissue, whereas the low biodegradability of PCL could avoid the collapse of the biomimetic trachea before the engineered tracheal cartilage is sufficient to hold the tracheal lumen. We envisaged that the PCL rings could be gradually replaced by host connective tissue (e.g., smooth muscle fibers) as the degradation of PCL, and eventually formed a biomimetic trachea with C-shaped cartilage rings made of cartilage connected at its open ends by smooth muscle fibers as well as alternating with connective tissue rings.

Finally, we investigated the feasibility of using the proposed biomimetic trachea to repair long-segment tracheal defects in an animal model to provide direct evidence of its potential for future clinical application. Although some authors demonstrating anatomically correct tracheal scaffolds, they didn’t achieved the repair of long-segment tracheal defects (Best et al., 2018; Townsend et al., 2018). Our results demonstrated that the biomimetic trachea could enable the survival and normal function in rabbits with artificial defects for at least 8 weeks post-surgery. Notably, in the current study, we deliberately avoided the use of a support to demonstrate that the proposed biomimetic trachea was sufficient to avoid the collapse of the tracheal lumen under the negative pressure during inspiration. The PCL framework was considered to be a vital factor contributing to the appropriate radial rigidity before the maturation of the cartilage ring. We speculate that the progressively matured cartilage ring will supplement the radial rigidity as the PCL scaffold degrades gradually beyond 8 weeks post-surgery. Although many previous studies have also achieved long-segment tracheal defect repair, most of those studies used a tracheal stent to prevent stenosis and resist the collapse of the trachea substitute. However, tracheal stents are associated with inflammation, re-stenosis, implant migration, tracheal obstruction, and hemorrhage (Ernst et al., 2007; Wurtz et al., 2010; Huang et al., 2016; Ahn et al., 2019). In addition, removable silicone stents hinder curative treatment and may induce granulation, especially in the subglottic region. Consequently, we demonstrated that the proposed biomimetic trachea serves as a tracheal replacement without the aid of a tracheal stent, making it a promising candidate for future clinical application.

Although the current study demonstrated the feasibility of the proposed biomimetic trachea in repairing long-segment tracheal defects, there were some limitations to this study that could be addressed in future studies. First, the long-term (i.e., beyond 8 weeks) performance of the tracheal repair needs to be further investigated. In addition, the proposed design should be tested in a large animal model that more closely mimics the human physiological condition (such as pigs or sheep). Future studies should focus on optimizing the source of seeded cells (for example, stem cells derived from bone marrow or adipose tissue could be used) and developing a technique to promote rapid reepithelization.

## Conclusion

In summary, this study presents a novel design and fabrication technique for a biomimetic tracheal scaffold with a separated-ring structure that mimics both the anatomy and mechanical characteristics of the native trachea using a 3D-printed PCL framework and with embedded collagen. Experimental results demonstrated the successful formation of a biomimetic trachea with substantial tracheal cartilage and its use in repairing a long-segment tracheal defect. This work represents a step toward solving the challenges and clinical limitations in treating tracheal lesions.

## Data Availability Statement

The original contributions presented in the study are included in the article/Supplementary Material, further inquiries can be directed to the corresponding author/s.

## Ethics Statement

All animal experiments were approved by the Shanghai Pulmonary Hospital Ethics Committee.

## Author Contributions

YS: culture cells and editing. ZF and LW: scaffolds design. YL and WS: revision and SEM test. HT and LZ: animal operation. LW: editing. HZ: mechanical test. CC: funding acquisition and review and editing. All authors contributed to the article and approved the submitted version.

## Conflict of Interest

The authors declare that the research was conducted in the absence of any commercial or financial relationships that could be construed as a potential conflict of interest.

## Supplementary Material

Supplementary Video 1 Radial rigidity of the biomimetic PCL scaffold.

Supplementary Video 2 Longitudinal flexibility of the biomimetic PCL scaffold.

Supplementary Video 3 Radial rigidity of the engineered biomimetic trachea cultured *in vivo*.

Supplementary Table 1 Postoperative evaluation of rabbits.
