# Supplementary material for: 3D Printed Biomimetic PCL Scaffold as Framework Interspersed With Collagen for Long Segment Tracheal Replacement
Source: Front Cell Dev Biol. 2021 Jan 21;9:629796. doi: 10.3389/fcell.2021.629796 (PMC7859529; doi:10.3389/fcell.2021.629796)
Supplement: Supplementary Table 1 — Postoperative evaluation of rabbits. [file Table_1.docx]

**Supplemental Materials**

**3D printed biomimetic PCL scaffold as framework interspersed with collagen for long segment tracheal replacement**

**Yunlang She^a^, Ziwen Fan^a^, Long Wang, Yinze Li, Weiyan Sun, Hai Tang, Lei Zhang, Liang Wu^*^, Hui Zheng^*^, Chang Chen^*^**

Department of Thoracic Surgery, Shanghai Pulmonary Hospital, Tongji University School of Medicine, Shanghai, P.R. China

^a^ These authors are equal contributors to this work

^*^Corresponding authors: Dr. Liang Wu, Email: [wuliang198209@yahoo.com](mailto:wuliang198209@yahoo.com); Dr. Hui Zheng, Email: [zh981117@126.com](mailto:zh981117@126.com); Dr. Chang Chen, Email: [changchenc@tongji.edu.cn](mailto:changchenc@tongji.edu.cn).

**Supplemental Table 1** Postoperative evaluation of rabbits

|  | Survival rate | Dehiscence | Leakage or collapse | Stridor or dyspnea | Pneumonia | Stenosis |
| --- | --- | --- | --- | --- | --- | --- |
| 4 weeks | 100% | None | None | None | None | Minor |
| 8 weeks | 100% | None | None | None | None | None |
